# Similarities and differences in the clinical features and management of primary lymphedema and kaposiform hemangioendothelioma associated with lymphedema in children

**DOI:** 10.3389/fped.2025.1480213

**Published:** 2025-02-14

**Authors:** Yujia Zhang, Tong Qiu, Congxia Yang, Jiangyuan Zhou, Min Yang, Xue Gong, Zixin Zhang, Yuru Lan, Xuepeng Zhang, Siyuan Chen, Yi Ji

**Affiliations:** ^1^ Department of Pediatric Surgery, West China Hospital, Sichuan University, Chengdu, China; ^2^ Med-X Center for Informatics, Sichuan University, Chengdu, China; ^3^ Department of Critical Care Medicine, West China Hospital, Sichuan University, Chengdu, China

**Keywords:** primary lymphedema, kaposiform hemangioendothelioma, clinical characteristics, complications, treatment

## Abstract

**Background:**

Primary lymphedema (PLE) and kaposiform hemangioendothelioma-related lymphedema (KLE) are rare vascular anomalies (VAs). This study aimed to examine the clinical features, management, and prognosis of PLE and KLE.

**Method:**

The clinical features, imaging, treatments, and outcomes of 12 patients with PLE and 12 patients with KLE were retrospectively reviewed.

**Results:**

The mean age at which signs/symptoms were diagnosed was 68.2 months for PLE patients and 25 months for KLE patients. In PLE, the involvement of multiple sites is common, whereas in KLE, it typically affects a single site. Morbid obesity, which is common in adult patients, is rare in pediatric PLE and KLE patients. Imaging agent accumulation was observed in KLE but not in PLE via lymphoscintigraphy. In contrast, complications of PLE primarily involve skin and soft tissue, whereas musculoskeletal system complications are more common in KLE. Regarding prognosis, most patients stabilize or even experience lesion regression after standard treatment.

**Conclusion:**

PLE and KLE share clinical symptoms. PLE often involves multiple sites, whereas KLE typically presents unilaterally with local lymphatic stasis. Standardized treatment enables the majority of children with lymphedema to control the disease without progression, with KLE showing potential reversibility. Given their rarity, a multidisciplinary approach is crucial for diagnosis and management.

## 1 Introduction

Lymphedema, an uncommon childhood vascular anomaly (VA), manifests as localized tissue swelling due to lymphatic drainage malfunction stemming from diverse causes. This malfunction leads to lymphatic system overload, inadequate lymph clearance, and the retention of lymphatic fluid beneath the skin (1–4). From an etiological perspective, lymphedema can be categorized into primary and secondary forms.

Primary lymphedema (PLE), which may present as an isolated symptom or as part of complex syndromes, primarily results from congenital malformations or underdevelopment of lymphatic vessels due to genetic mutations (1). Secondary lymphedema (SLE) occurs due to damage or obstruction of previously normal lymphatic vessels caused by disease progression, infection, trauma, surgery, obesity, malignancy, or therapeutic interventions related to malignancy, such as lymph node dissection and radiation therapy. Additionally, complex vascular malformations are associated with the development of lymphedema. Genetic susceptibility may also contribute to the development of SLE (5). In pediatric patients, PLE predominates, accounting for 97% of cases (4), whereas SLE is relatively rare.

Owing to dysregulated angiogenesis and lymphangiogenesis, kaposiform hemangioendothelioma (KHE) is another rare VA that occurs mainly in the pediatric population (6–8). Several studies have indicated that lymphedema is a complication of KHE, which can lead to dysfunction, infections, chronic skin changes, and psychosocial disorders in severe cases (6, 9). Few studies are comparing these two distinct types of lymphedema. Considering the rarity and clinical similarities between PLE and kaposiform hemangioendothelioma-related lymphedema (KLE), our study described and analyzed the clinical manifestations, radiological characteristics, associated complications, treatments, and prognoses of these two disorders. This study aims to deepen the understanding of these distinct types of lymphedema and offer insights for future medical research.

## 2 Methods

### 2.1 Patients and data collection

This retrospective study received approval from the Institutional Review Board of West China Hospital of Sichuan University. All procedures adhered to the study protocol sanctioned by both West China Hospital at Sichuan University and Sichuan University and were conducted in compliance with the principles outlined in the Declaration of Helsinki.

The study population included patients with PLE and patients with KLE. All patients were diagnosed and followed up at our medical center for 6 years from July 2017 to September 2023. The diagnosis of PLE and KLE was based on clinical features, physical examination [e.g., Stemmer's sign (10)], magnetic resonance imaging (MRI), lymphoscintigraphy, and/or histological data (1–5, 11, 12), as well as the results of a multidisciplinary consultation on VAs at West China Hospital of Sichuan University. Patients whose age of onset was later than 18 years or whose data were insufficient were excluded.

Patient outcomes were collected through clinical follow-up and telephone interviews, with the last follow-up conducted in December 2023. The researchers evaluated patients' demographics, clinical presentations, imaging findings, extremity swelling, treatment modalities, associated complications, and follow-up outcomes via a combination of interviews and medical records review. The classification of childhood obesity and overweight across different age and sex groups was determined based on body mass index (BMI), in accordance with national health standards outlined in the “Growth Standard for Children Under 7 Years of Age” (13) and “Screening for Overweight and Obesity Among School-Age Children and Adolescents” (14). A decreased range of motion (ROM) was defined as extension loss of ≥10°, flexion <120°, or both (15).

### 2.2 Statistical analysis

We used IBM SPSS Statistics 25.0 software for data calculation. Categorical data are expressed as *n* (%), and quantitative data are presented as means (ranges). Fisher's exact test, *t*-test, or the Mann−Whitney *U* test were used appropriately. Multivariate logistic regression analysis was employed to adjust for the effects of variables such as age, sex, BMI, and other clinical factors that could influence the outcomes. Matching techniques were also utilized where applicable to ensure that cases and controls were comparable with respect to key demographic and clinical characteristics. This approach allowed for the calculation of adjusted odds ratios (ORs). *P*-values <0.05 were considered statistically significant, and the OR and 95% confidence intervals (95% CI) were calculated.

## 3 Results

### 3.1 Baseline characteristics

In total, 24 patients were included in the study. A summary of the patients is provided in Table 1. Twelve of these 24 patients were diagnosed with PLE. The mean age at the time of presentation of the signs and/or symptoms of PLE was 35.5 months (range, 0–110 months), whereas that at the time of diagnosis was 68.2 months (range, 5–150 months). There were nine males and three females.

**Table 1:** Demographic and clinical characteristics of patients with PLE and KLE.

Variables	PLE (*n* = 12)	KLE (*n* = 12)	*P*-value	Odds ratio (95%CI)
Patients				
Gender^a^ (*n*, %)			1^b^	1.500 (0.254–8.844)
Male	9 (75)	8 (67)		
Female	3 (25)	4 (33)		
Age at the time of the presentation of the signs and/or symptoms (months)			0.352^c^	N/A
Mean (range)	35.5 (0–110)	19.8 (0–59)		
Age at diagnosis (months)		0.008^d^	N/A	
Mean (range)	68.2 (5–150)	25 (0–59)		
Involvement^a^ (*n*, %)			0.322^b^	
Upper limbs	3 (25)	4 (33)		1.091 (0.230–5.185)
Lower limbs	11 (91.7)	8 (67)		2.333 (0.413–13.171)
Perineum	3 (25)	0 (0)		N/A
Distribution^a^ (*n*, %)			0.014^b^	N/A
Single-site	6 (50)	12 (100)		
Multisite	6 (50)	0 (0)		
Body mass index (kg/m^2^)		0.366^d^	N/A	
Mean (range)	16.9 (11.6–22)	16.1 (13.3–19.5)		
Nutriture (*n*, %)			1^b^	1.500 (0.254–8.844)
Normal weight^a^	8 (67)	9 (75)		
Overweight^a^	4 (33)	3 (25)		

PLE, primary lymphedema; KLE, kaposiform hemangioendothelioma-related lymphedema; CI, confidence interval; N/A, data not available.

^a^ Values are presented as the number of cases (percentage).

^b^ *P*-value is calculated using *t*-test.

^c^ *P*-value is calculated using the Mann‒Whitney *U* test.

^d^ *P*-value is calculated using Fisher's exact test.

KLE was reported in 12 patients, including eight males and four females. The mean age at the time of the presentation of signs and/or symptoms was 19.8 months (range, 0–59 months). The mean age at diagnosis of KLE was 25 months (range, 0–50 months). There was no statistically significant difference in the age of onset between patients with PLE and those with KLE. However, it is notable that the age at diagnosis for patients with KLE was significantly earlier than that for those with PLE (*P* = 0.008).

### 3.2 Clinical symptoms

After analyzing the engagement of all 24 patients, it was observed that half of the patients in the PLE group exhibited involvement at more than one site, with 11 patients (91.7%) experiencing lymphedema of the lower limbs and 3 patients presenting with perineal involvement. Conversely, within the KLE group, all patients demonstrated involvement at a single site.

We assessed the growth and development of the patients based on BMI. The mean BMI of patients in the PLE group was 16.9 kg/m^2^ (range, 11.6–22 kg/m^2^), whereas that of patients in the KLE group was 16.1 kg/m^2^ (range, 13.3–19.5 kg/m^2^). Four patients (33.3%) with PLE and three patients (25%) with KLE were assessed as overweight.

### 3.3 Lymph scintigraphy

All patients underwent lymphoscintigraphy, revealing impaired lymphatic reflux in 23 patients (95.8%), with 12 in the KLE group and 11 in the PLE group. The results are shown in Table 2. Among the patients in the PLE group, five patients (41.7%) exhibited minimal radiolabeled colloid accumulation in the axillary/inguinal lymph nodes on the affected side 3 h postinjection, which was significantly less than that in the contralateral normal limb. Six patients (50%) experienced lymphatic obstruction, with negligible drainage from the axillary/inguinal lymph nodes on the affected side, whereas four patients (33.3%) displayed dermal backflow, indicating tracer accumulation in the dermal lymphatics. Additionally, findings from one patient suggested normal lymphatic reflux.

**Table 2:** Lymphoscintigraphic findings of patients with PLE and KLE.

Variables	PLE	KLE
	*n* = 12	*n* = 12
Lymphoscintigraphic findings^a^		
Normal lymphatic flow	1 (8.3)	0 (0)
Dermal backflow	4 (33)	1 (8.3)
Delayed lymphatic drainage	5 (41.7)	3 (25)
Lymphatic obstruction	6 (50)	9 (75)
Lymphatic stasis	0 (0)	12 (100)

PLE, primary lymphedema; KLE, kaposiform hemangioendothelioma-related lymphedema.

^a^ Values are presented as the number of cases (percentage).

Among the 12 patients in the KLE group, only 1 patient experienced dermal backflow, 3 patients (25%) experienced delayed lymphatic drainage, and 9 patients (75%) developed lymphatic obstruction. Furthermore, all patients in the KLE group exhibited varying degrees of lymphatic stasis, with the affected site corresponding to the primary lesion of KHE.

### 3.4 Complications

**Figure 1:**
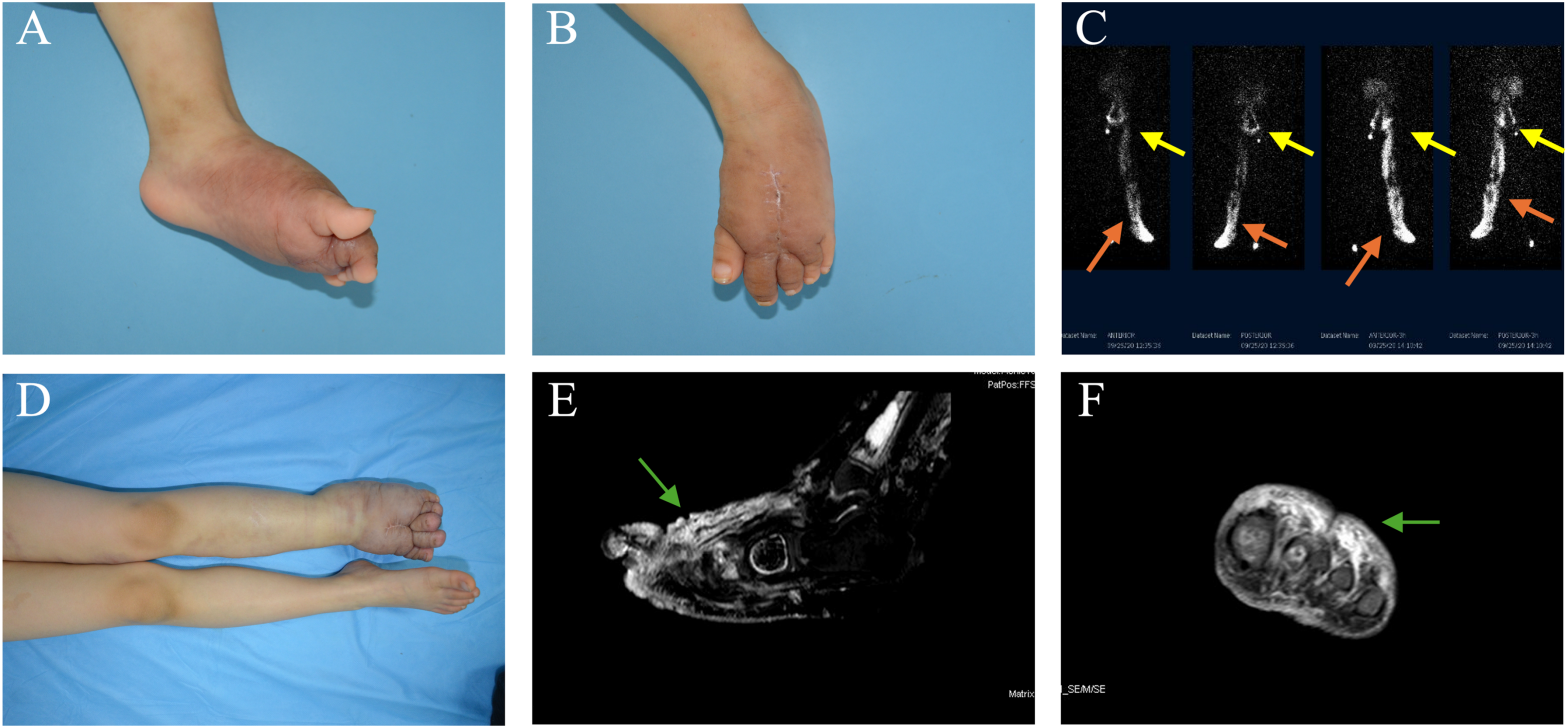
A female patient with KHE affecting the left foot presented with lymphedema. KHE onset occurred at 2 years of age, and limb swelling occurred 2 years later, at the age of 4. **(A**,**B)** Photographs taken at the time of referral depict swelling of the left foot, along with an old surgical scar on the dorsum of the foot. **(C)** Radionuclide lymphoscintigraphy revealed uptake in the bilateral inguinal and pelvic lymph nodes at 100 and 150 min after injection into the left foot (yellow arrows). Abnormal tracer accumulation and dermal reflux were observed in the left lower limb (marked by the concentrated white area, as shown by the orange arrows). **(D)** After 33 months of combined drug therapy and physical therapy, worsening swelling was observed in the left foot. **(E,F)** Sagittal and coronal T2-weighted images revealed high-intensity lesions in the dermis, subcutaneous tissue, and deep fascia of the left foot, accompanied by soft tissue swelling around the lesions (green arrows).

The results of the complications are shown in Table 3. Cellulitis occurred in one patient (91.7%) in each of the PLE and KLE groups. Three patients (75%) in the PLE group developed cutaneous problems (e.g., lymphorrhagia and ulceration), whereas those in the KLE group did not exhibit similar symptoms. Orthopedic complications exclusively occurred in the KLE group, with three patients (75%) experiencing varying degrees of decreased range of motion and two patients (16.7%) developing bone destruction (Figure 1).

**Table 3:** Management and outcomes of patients with PLE and KLE.

Variables	PLE	KLE
	*n* = 12	*n* = 12
Therapy methods^a^^,^^b^		
Sirolimus	1 (8.3)	12 (100)
Physical therapy	10 (83.3)	10 (83.3)
Surgery	1 (8.3)	5 (41.7)
None	2 (16.7)	0 (0)
Outcome^a^		
Improved	4 (33)	4 (33)
Stable	7 (58.3)	7 (58.3)
Worse	1 (8.3)	1 (8.3)
Complications^a^		
Decreased range of motion	0 (0)	3 (25)
Bone destruction	0 (0)	2 (16.7)
Cellulitis	1 (8.3)	1 (8.3)
Cutaneous (lymphatic vesicles, ulceration)	3 (25)	0 (0)

PLE, primary lymphedema, KLE, kaposiform hemangioendothelioma-related lymphedema.

^a^ Values are presented as the number of cases (percentage).

^b^ Some patients received more than one treatment modality.

### 3.5 Treatments and outcomes

All 24 patients were enrolled in the follow-up study. The patients in this study received various treatments, including medication, physiotherapy, and surgery. The physiotherapy regimen included the use of compression garments, compression bandaging, and manual lymphatic drainage (MLD). Depending on the location of the lymphedema, patients wore appropriate compression clothing: stockings for the lower limbs, glove sleeves for the upper limbs, and compression shorts tailored to the perineum. Compression garments were used in conjunction with compression bandaging to exert additional pressure on the affected area. Furthermore, due to the logistical challenges faced by many patients from other provinces, who found it difficult to attend regular MLD sessions at the clinic, our professional lymphedema physiotherapists provided training to the patients' parents. This enabled the parents to administer MLD treatment at home, thereby ensuring continuity of care.

**Figure 2:**
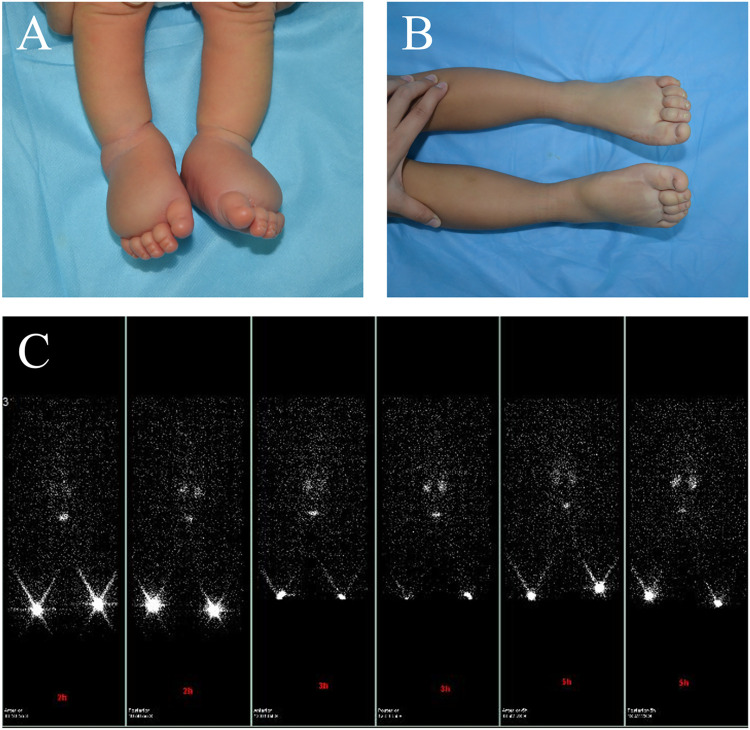
A male patient presented with primary lymphedema affecting both feet. The patient's feet were noted to be swollen during prenatal examination. **(A)** At birth, the patient exhibited swelling in the feet, and the Stemmer sign was positive. **(B)** After 3 years of physical therapy, the swelling improved. **(C)** Bilateral lower limb lymphoscintigraphy at age 3 revealed that the inguinal lymph nodes on both sides did not show any uptake.

Among these individuals, 14 (58.3%) maintained stability in their condition (Figure 2), 2 (8.3%) experienced deterioration, and 8 (33.3%) experienced improvement. In the PLE group, 10 (83.3%) patients received physiotherapy, and 1 (8.3%) patient was additionally prescribed sirolimus medication for 1 year. Conversely, all patients in the KLE group were administered oral sirolimus treatment, with 10 (83.3%) patients among them also receiving combined physiotherapy.

**Figure 3:**
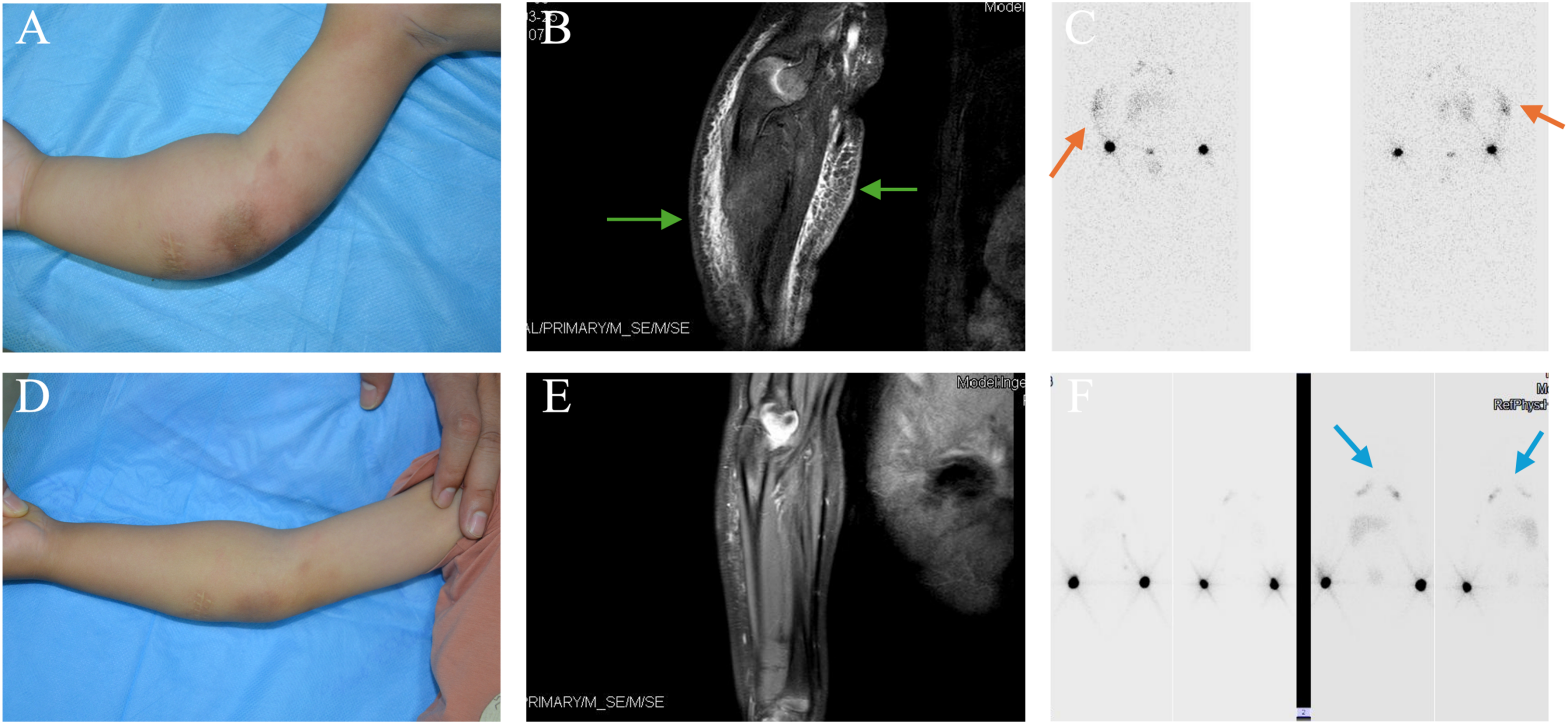
KHE-associated lymphedema affected the right upper extremity in a male patient. Vascular lesions were observed in this patient at birth. Limb swelling occurred 19 months later. **(A)** The photograph depicts tight swelling on the right arm at referral (at 19 months of age). **(B)** Coronal T2-weighted MR image revealing hyperintense lesions affecting the dermis, subcutaneous tissue, and deep fascia of the right arm, accompanied by swelling of the surrounding soft tissue (appearing as a blurry dark area, as shown by the green arrows). **(C)** The nuclear lymphoscintigram acquired 100 and 150 min after injection into the right arm revealed tracer uptake in the left axillary lymph nodes but not on the right side. Notably, abnormal tracer accumulation was observed in the right elbow (orange arrows). **(D,E)** Fifteen months after compression and medication, the swelling of his right elbow was reduced, and MRI revealed fewer high-intensity lesions in the dermis, subcutaneous tissue, and deep fascia than in panel **B**. **(F)** At 34 months of age, lymphoscintigraphy revealed visualization of both sides of the axillary nodes 1 h after injection into the right limb (blue arrows), indicating normal lymphatic reflux of the right upper arm.

Among the cohort, six (25%) patients underwent various types of surgical procedures, all of which were performed prior to referral. Notably, five (41.6%) patients in the KLE group underwent surgery specifically aimed at addressing the primary disease. The symptoms of 1 KLE patient were relieved after receiving sirolimus combined with physiotherapy. Imaging revealed lymphatic flow (Figure 3).

## 4 Discussion

The study design involves a single-center retrospective analysis focusing on pediatric PLE and KLE. Our study revealed that patients with PLE and those with KLE presented comparable characteristics in terms of sex ratio, nutritional status, and treatment outcomes. Nonetheless, the duration between onset and diagnosis was shorter in the KLE group than in the PLE group. This disparity may be attributed to the primary lesion underlying this secondary lymphedema. Research indicates that the onset of KHE primarily occurs in infants under 1 year of age (16), with 11% of patients experiencing this complication after the first year of onset (5), whereas PLE can manifest in children across various age groups (17). PLE is primarily caused by congenital abnormalities in the development of the lymphatic system and is often associated with genetic mutations in FLT4, FOXC2, GJC2, and PIEZO1 (18–20). These genetic alterations disrupt the development and function of lymphatic vessels, leading to impaired lymphangiogenesis and compromised lymphatic function. While family aggregation has been observed in some patients with lymphedema, no such pattern was identified among the patients included in this study. In the case of KLE, analysis of lymphoscintigraphy and clinical manifestations reveals that KHE lesions themselves disrupt local lymphatic drainage. Lymphatic dysplasia is the primary pathological feature of both KHE and lymphedema (6, 7, 21, 22). Additionally, KHE-derived mesenchymal stem cells not only express vascular endothelial growth factor receptor 3 (VEGFR3) but also exhibit significantly higher levels of vascular endothelial growth factor C (VEGF-C) than normal lymphoendothelial cells do (23). The VEGF-C/VEGFR3 signaling pathway plays a critical role in this process (2, 24–26). Through the activation of downstream pathways such as the PI3K/AKT and RAS/MAPK pathways, this signaling cascade promotes the proliferation and migration of lymphatic endothelial cells, which are essential for normal lymphatic development (27, 28).

Interestingly, we have observed clinically that some children with PLE, particularly those presenting with bilateral limb involvement, may not exhibit overt symptoms during the early stages of the disease. Parents become aware of this phenomenon only when they encounter traumatic or inflammatory stimuli, such as respiratory infections, which precipitate rapid deterioration of the disease within a short timeframe. This finding aligns with the findings of other researchers suggesting that inflammation may serve as a primary stimulant for the progression of lymphedema. Inflammatory mediators such as IL-4, IL-13, TGF-β, and LTB4 are implicated in various pathways that promote lymphedema progression (29–31). Furthermore, inflammation associated with the Kasabach–Merritt phenomenon (KMP) may result in obstruction and retrograde flow of multiple lymphatic vessels (7).

Consistent with findings from previous studies (4, 32, 33), the majority of patients exhibited lower extremity PLE, often with bilateral involvement, in contrast with the unilateral involvement typically observed in the KLE group. Importantly, the lymphedema site in patients in the KLE group in our study was correlated with the site of KHE. Interestingly, upon examination via lymphoscintigraphy, we observed imaging agent stasis at the lesion in all KLE patients, which was indicative of locally impaired lymphatic drainage. These findings indicate that lymphedema occurring within the same lymphatic drainage area as KHE does not manifest independently of KHE.

Obesity has been proposed as a risk factor for the onset of lymphedema. Increased local adipose tissue can obstruct lymphatic vessels in the drainage area (34, 35). A BMI of >60 kg/m^2^ can impair lymphatic function (36), while lymphedema can induce fat deposition, establishing a vicious cycle and contributing to the strong association between lymphedema and obesity (9). Given the early onset and limited follow-up of lymphedema in children compared with adult cohorts and the considerable time it may take for associations between lymphedema and obesity to become apparent, our study included a low proportion of overweight patients, most of whom had a BMI within the normal range. Considering the detrimental impact of obesity on lymphedema progression, clinicians should prioritize weight management in the treatment of such children to mitigate the risk of disease exacerbation.

Complications in patients with PLE affect various systems, with cellulitis being the most prevalent, followed by dermatological issues such as ulcers and lymphorrhagia, whereas motor system-related lesions are relatively infrequent (4, 36, 37). Conversely, a larger proportion of patients with KHE presented musculoskeletal lesions (6, 8, 15). Platelet activation and aggregation within the lesion can induce fibrosis, whereas fibrosis surrounding the joint can exacerbate muscle atrophy, consequently restricting joint mobility (38). Additionally, excessive limb swelling caused by lymphedema can result in abnormal movement of the affected limb. In this study, four patients with PLE developed cellulitis and skin-related complications, whereas musculoskeletal complications were observed in only five patients in the KLE group. Among them, three patients achieved control with sirolimus adjunctive to physiotherapy.

Despite variations in the etiology and severity of lymphedema in children, numerous studies have indicated that physiotherapy is the primary approach to lymphedema treatment (39–41). Furthermore, the management of KLE should be synchronized with KHE treatment, utilizing oral sirolimus, which is recognized as the primary therapeutic agent for KHE treatment (42–44).

In this study, patients in both the KLE and PLE groups demonstrated comparable outcomes in terms of prognosis, with a non-inferiority evaluation rate of 91.7%. Notably, in one KLE patient in this study, following 15 months of physiotherapy alongside sirolimus, limb swelling markedly diminished. Lymphoscintigraphy revealed unobstructed lymphatic vessels, leading to complete disease remission, suggesting that chronic lymphedema stemming from KHE may not be irreversible. Sirolimus, an mTOR inhibitor, has emerged as a first-line treatment for KHE due to its ability to block the mTOR signaling pathway (45). It downregulates the expression of VEGF-A/VEGFR-2 and VEGF-C/VEGFR-3, reduces the production of various cytokines, and inhibits tumor angiogenesis by decreasing both angiogenesis and lymphangiogenesis.

While earlier studies speculated that sirolimus treatment might contribute to lymphedema (46), our prior research demonstrated that a history of sirolimus use is not a risk factor for lymphedema (5). In fact, some patients with sirolimus-associated lymphedema have shown improvement or resolution of symptoms following discontinuation of the drug. Furthermore, sirolimus has been shown in animal models to inhibit the growth of abnormal lymphatic vessels and reduce lesions associated with lymphatic dilation without adversely affecting normal lymphatic vessels (47). Sirolimus has strong antiproliferative, antiangiogenic, and antilymphangiogenic effects, as well as proapoptotic and autophagic effects, on various vascular malformations. When combined with physical therapy, sirolimus may represent a promising therapeutic approach for managing lymphedema in children.

Consequently, despite the challenge of detecting lymphedema due to limb swelling induced by KHE and KMP, clinicians should remain vigilant and administer timely symptomatic treatment. Furthermore, considering the influence of inflammation on lymphedema observed in this study, previous research has indicated that targeted anti-inflammatory therapy can enhance disease management in terms of histopathology and skin thickness (29, 31, 32, 48). In addition to conventional physiotherapy, clinicians should assess the inclusion of anti-inflammatory therapy in medical decision-making processes.

## 5 Limitations

First, owing to its single-center retrospective nature, the sample size is limited, and systematic bias is inevitable. Because our study subjects were minors, the degree of parental attention may have caused selection bias. Second, our patients received various treatment regimens before referral to our center, and we cannot rule out the existence of a discernible effect of these measures on the development of lymphedema. As this is the first study to compare these two forms of lymphedema, the strengths of this study include that it provides valuable information concerning rare diseases. Finally, this was an observational study, and the mechanism of this type of disease, especially the cause of lymphatic abnormalities, should be further investigated in the future.

## 6 Conclusion

To provide a better understanding of these rare diseases, our study describes two distinct types of lymphedema, detailing their clinical presentations and comparing various features. While PLE and KLE share some clinical symptoms, PLE often involves multiple sites, whereas KLE typically presents in the limb ipsilateral to the primary lesion, accompanied by local lymphatic stasis evident via lymphoscintigraphy. Standardized treatment allows the majority of children with lymphedema to control the disease without progression. Our study underscores the importance of early diagnosis and sustained treatment initiation for this chronic disease. Given the rarity of PLE and KLE, a multidisciplinary approach is indispensable for the diagnosis and long-term management of these conditions.

## Data Availability

The original contributions presented in the study are included in the article/Supplementary Material, further inquiries can be directed to the corresponding author.
